# Noncanonical Mismatch Repair Protein NucS Modulates the Emergence of Antibiotic Resistance in Mycobacterium abscessus

**DOI:** 10.1128/spectrum.02228-22

**Published:** 2022-10-11

**Authors:** Rosilene Fressatti Cardoso, Isabel Martín-Blecua, Vanessa Pietrowski Baldin, Jean Eduardo Meneguello, José Ramón Valverde, Jesús Blázquez, Alfredo Castañeda-García

**Affiliations:** a Departamento de Análises Clínicas e Biomedicina, Universidade Estadual de Maringá, Maringá, Paraná, Brazil; b Departamento de Biotecnología Microbiana, Centro Nacional de Biotecnología CNB-CSIC, Madrid, Spain; c Departamento de Computación Científica, Centro Nacional de Biotecnología CNB-CSIC, Madrid, Spain; Johns Hopkins University School of Medicine

**Keywords:** *Mycobacterium abscessus*, drug resistance, antibiotic resistance, NucS, EndoMS, mutation, acquired resistance, mismatch repair, noncanonical mismatch repair, DNA repair, macrolides, aminoglycosides

## Abstract

NucS/EndoMS-dependent noncanonical mismatch repair (MMR) ensures the stability of genomic DNA in mycobacteria and acts as a guardian of the genome by preventing the accumulation of point mutations. In order to address whether the inactivation of noncanonical MMR could increase the acquisition of drug resistance by mutation, a Δ*nucS* strain was constructed and explored in the emerging pathogen Mycobacterium abscessus. Deletion of *nucS* resulted in a mutator phenotype with increased acquisition of resistance to macrolides and aminoglycosides, the two main groups of antimycobacterial agents for M. abscessus treatment, and also to second-line drugs such as fluoroquinolones. Inactivation of the noncanonical MMR in M. abscessus led to increases of 10- to 22-fold in the appearance of spontaneous mutants resistant to the macrolide clarithromycin and the aminoglycosides amikacin, gentamicin, and apramycin, compared with the wild-type strain. Furthermore, emergence of fluoroquinolone (ciprofloxacin) resistance was detected in a *nucS*-deficient strain but not in a wild-type M. abscessus strain. Acquired drug resistance to macrolides and aminoglycosides was analyzed through sequencing of the 23S rRNA gene *rrl* and the 16S rRNA gene *rrs* from independent drug-resistant colonies of both strains. When the acquisition of clarithromycin resistance was examined, a different mutational profile was detected in the M. abscessus Δ*nucS* strain compared with the wild-type one. To summarize, M. abscessus requires the NucS-dependent noncanonical MMR pathway to prevent the emergence of drug-resistant isolates by mutation. To our knowledge, this is the first report that reveals the role of NucS in a human pathogen, and these findings have potential implications for the treatment of M. abscessus infections.

**IMPORTANCE** Chronic infections caused by M. abscessus are an emerging challenge in public health, posing a substantial health and economic burden, especially in patients with cystic fibrosis. Treatment of M. abscessus infections with antibiotics is particularly challenging, as its complex drug resistance mechanisms, including constitutive resistance through DNA mutation, lead to high rates of treatment failure. To decipher the evolution of antibiotic resistance in M. abscessus, we studied NucS-dependent noncanonical MMR, a unique DNA repair pathway involved in genomic maintenance. Inactivation of NucS is linked to the increase of DNA mutations (hypermutation), which can confer drug resistance. Our analysis detected increased acquisition of mutations conferring resistance to first-line and second-line antibiotics. We believe that this study will improve the knowledge of how this pathogen could evolve into an untreatable infectious agent, and it uncovers a role for hypermutators in chronic infectious diseases under antibiotic pressure.

## INTRODUCTION

The Mycobacterium abscessus complex is a group of nontuberculous mycobacteria (NTM) widely considered the most pathogenic and chemotherapy resistant among the rapidly growing mycobacteria (RGM) (1). NTM are opportunistic pathogens, as they are able to produce serious chronic pulmonary infections in susceptible patients infected from environmental sources (2) or previously infected patients (3, 4). NTM are a challenge in clinical settings due to the recent sharp rise of NTM infections, as these infections have become even more frequent than tuberculosis (TB) infections in developed countries (5).

M. abscessus is an emerging pathogen responsible for a wide variety of human diseases, including chronic pulmonary diseases and several extrapulmonary diseases such as skin, soft tissue, and bone infections (6). It is the most frequent RGM isolated from patients with structural lung diseases, including cystic fibrosis (CF), bronchiectasis, and chronic obstructive pulmonary disease, leading to severe infections in patients with chronic impairments in respiratory function (1). M. abscessus is considered a major emerging pathogen in CF, as it is able to promote lung function decline (7, 8), and it is a serious concern for lung transplantation (9).

M. abscessus exhibits intrinsic resistance to a wide variety of antibiotics, including the first-line anti-TB agents rifampin and isoniazid (10, 11). Clinical management of M. abscessus infection is challenging due to the limited number of effective drugs available and the limited success of prolonged chemotherapy (12). Only a few groups of antibiotics are active against this pathogen, with macrolides and aminoglycosides considered the most effective drugs for the treatment of M. abscessus infections (13, 14). A multidrug antibiotic treatment based on the combination of an oral macrolide (clarithromycin or azithromycin) and intravenous aminoglycoside (amikacin), occasionally with a second-line antibiotic (e.g., a β-lactam, quinolone, tetracycline, or oxazolidinone), is recommended for the treatment of M. abscessus infections (14–17). However, outcomes are generally poor in patients with chronic pulmonary infections, and refractory infections, relapse, and death are common (18).

Acquired resistance by genomic mutations is one of the main ways for the development of drug resistance to the limited available drugs, leading to constitutive resistance (1, 19). The emergence of drug-resistant M. abscessus clinical strains to macrolides and aminoglycosides is generated through the modification of the drug target, the ribosome. Acquired mutations in rRNA genes *rrl* (encoding the 23S rRNA) and *rrs* (encoding the 16S rRNA) are known to confer resistance to macrolides and aminoglycosides, respectively (20–22). A recent study of M. abscessus clinical isolates by genome sequencing found constitutive genotypic resistance mechanisms to macrolides and aminoglycosides in 7.3% (*rrl* mutations) and 4.3% (*rrs* mutations) of all the analyzed strains (4).

Intrinsic and inducible antibiotic and target-modifying enzymes are also key factors for resistance to most classes of antibiotics, including first-line macrolides and aminoglycosides, for M. abscessus (23–25). For instance, an Erm methylase encoded by a functional *erm*(41) gene modifies the 23S rRNA, generating induced resistance to macrolides and leading to chemotherapy failure with some clinical strains of M. abscessus (23, 26). The combination of constitutive and inducible resistance mechanisms can exacerbate the challenges of antibiotic efficacy in patients with M. abscessus infections (19).

Increased mutation rates (hypermutation), mostly due to impaired mismatch repair (MMR) capacity, are frequently observed in bacterial isolates from patients with chronic pulmonary infections, including CF (27–29). It is possible, therefore, that a common bacterial pathogen in CF patients, such as M. abscessus, could also develop a similar strategy to increase the acquisition of resistance through mutation. With limited genetic tools available, M. abscessus remains a challenging pathogen to explore deeply. It has recently been described that mycobacteria utilize an alternative noncanonical MMR pathway, based on the key DNA repair endonuclease NucS/EndoMS protein (30, 31). However, the role of this genome maintenance pathway in a human pathogen remains unknown. Here, we studied the association between NucS/EndoMS and the frequency of acquisition of antibiotic resistance mutations in M. abscessus by inactivating *nucS* through allelic exchange. The rates of mutations conferring resistance to a wide number of chemotherapeutic agents, including first-line antibiotics, were analyzed to reveal the impact of NucS/EndoMS inactivation on the emergence of drug-resistant M. abscessus isolates and to speculate on its putative role in the development of resistance in clinical settings.

## RESULTS

### Identification and characterization of M. abscessus *nucS*.

NucS was previously characterized as a DNA repair endonuclease able to specifically recognize and cleave mismatched DNA substrates (32–35), supporting a core role as an alternative MMR pathway. We previously identified that Mycobacterium smegmatis NucS/EndoMS has an essential function in maintaining genomic DNA stability, as inactivation of the *nucS* gene led to a strong increase in the rate of spontaneous mutations (30, 31).

**FIG 1:**
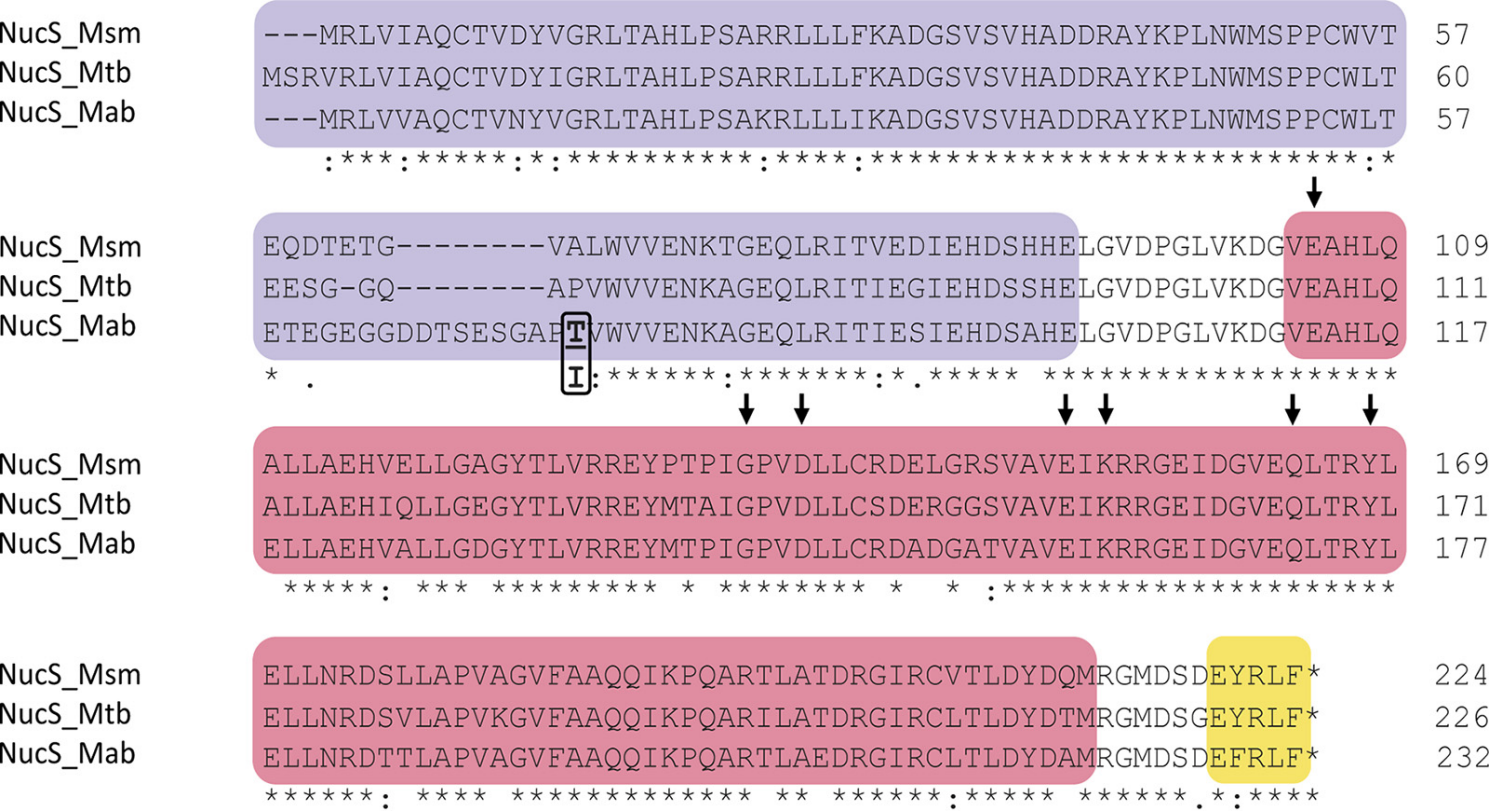
Multiple sequence alignment of the NucS protein sequences in mycobacteria. The alignment shows NucS sequences of M. smegmatis mc^2^ 155 (NucS_Msm), M. tuberculosis H37Rv (NucS_Mtb), and M. abscessus ATCC 19977 (NucS_Mab). Colors indicate protein domains according to the NucS structure: DNA-binding domain (purple), catalytic domain (pink), and β-clamp binding sequence (yellow). Symbols beneath the sequences: asterisks indicate identical amino acids, a colon indicates conservation between groups of strongly similar properties, and a period indicates conservation between groups of weakly similar properties. Arrows indicate key catalytic residues required for nuclease activity. The amino acid substitution found in some M. abscessus clinical strains by bioinformatics analysis is represented in bold in a square.

To identify the *nucS* gene in M. abscessus, the M. smegmatis NucS protein sequence was compared to the proteins encoded by the M. abscessus ATCC 19977 genome by using BLASTp (NCBI). The MAB_1460 (*nucS_Mab_*) gene was detected as a *nucS* ortholog, encoding a conserved protein that showed 83% identity to NucS proteins of both M. smegmatis and M. tuberculosis (Fig. 1). All of the key residues essential for DNA cleavage were conserved in the catalytic domain of M. abscessus NucS, in accordance with its function as a DNA repair endonuclease (Fig. 1).

**FIG 2:**
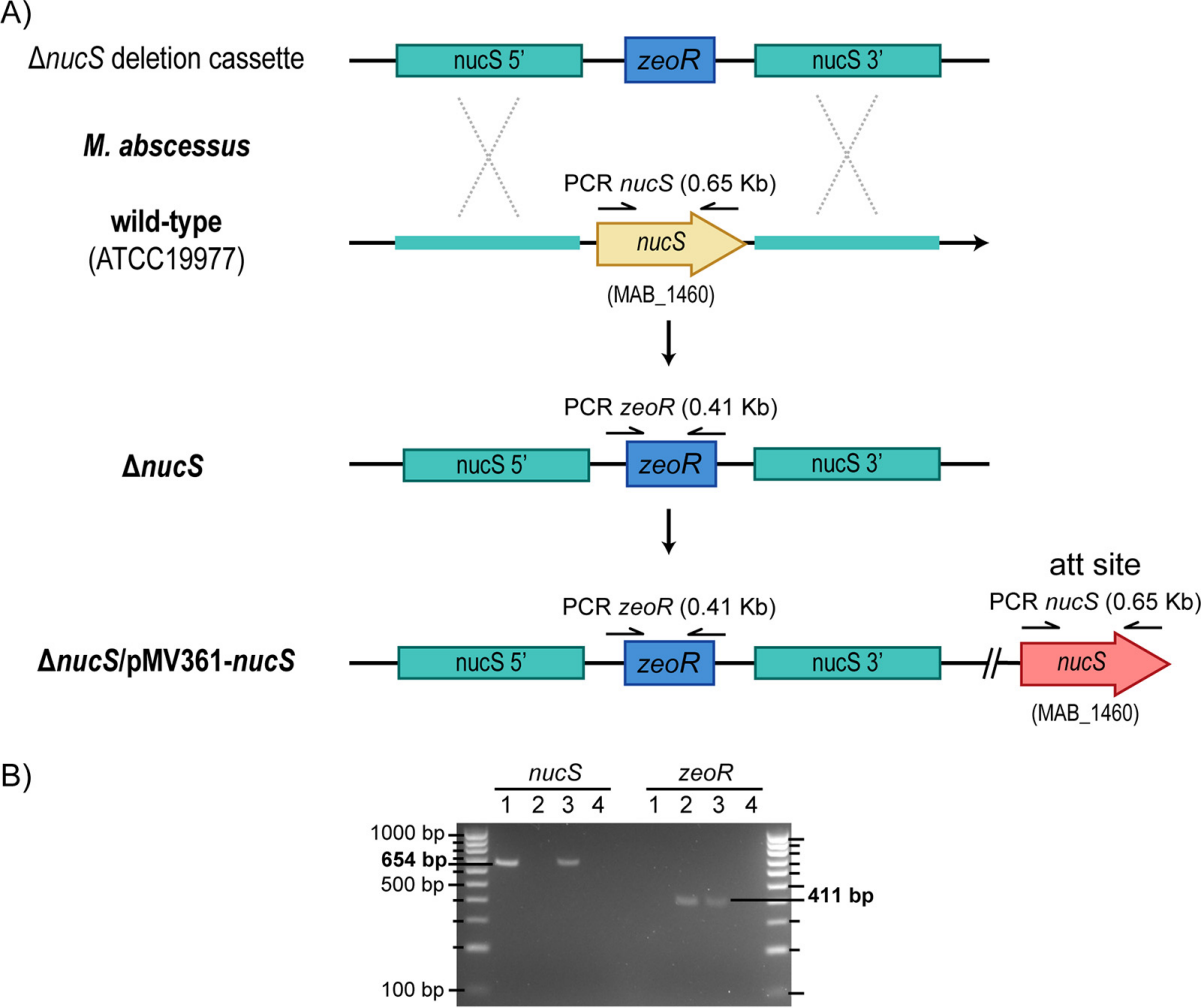
Construction and verification of M. abscessus strains. (A) Genetic scheme for the deletion of the *nucS* target gene in M. abscessus by recombineering (double recombination) with a Δ*nucS* deletion cassette and complementation with a wild-type *nucS* copy. (B) Gel electrophoresis analysis showing PCR products of *nucS* (left) and *zeoR* (zeocin resistance gene) (right). Lanes: 1, wild-type strain; 2, Δ*nucS* strain; 3, complemented strain; 4, negative control.

To characterize the function of M. abscessus NucS, a *nucS*-deficient strain was constructed by deleting the target gene through gene replacement (Fig. 2A). The limited availability of genetic tools to generate allelic exchange mutants in M. abscessus has complicated deeper genetic studies in this pathogen (36, 37). A *nucS_Mab_* gene disruption was generated by a double-crossover through recombination using the recombineering technique (38). The deletion plasmid was generated by cloning two flanking fragments (*nucS_Mab_* upstream and downstream regions) in a backbone vector (pSGV53) (39) containing a zeocin resistance gene. The knockout mutant was generated when the PCR-amplified cassette containing the gene deletion was inserted in the chromosome, replacing the *nucS_Mab_* gene with an antibiotic marker (Fig. 2A) (see Materials and Methods). Once M. abscessus Δ*nucS* was isolated and confirmed, a complemented strain was obtained through the integration of a wild-type *nucS* copy in its genome by site-specific recombination at the *att* site (Fig. 2A). Specific PCRs were examined to verify the deletion of the *nucS* gene in the M. abscessus Δ*nucS* strain and the integration of a *nucS* copy in the M. abscessus complemented strain (Fig. 2B).

Growth curves were performed to characterize the bacterial growth and viability of the M. abscessus wild-type and Δ*nucS* strains. The analysis of the growth curves did not reveal any significant differences in the growth rates and/or viability between the wild-type strain and the Δ*nucS* strain (see Fig. S1 in the supplemental material).

### M. abscessus mutation rates to first-line antibiotics.

**FIG 3:**
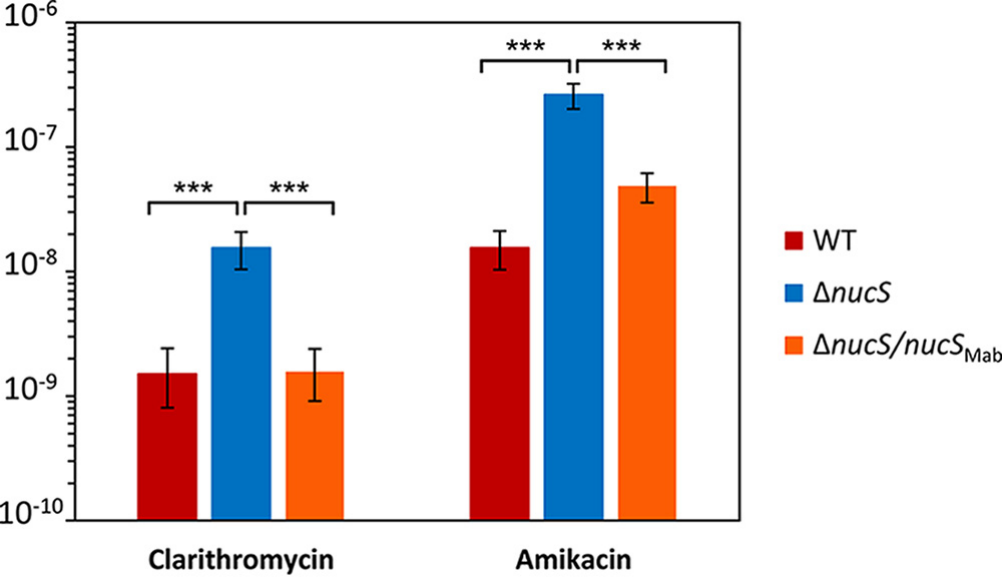
M. abscessus mutation rates to clarithromycin and amikacin. Rates of spontaneous mutations conferring antibiotic resistance are shown for M. abscessus ATCC 19977 (wild type [WT]; red), its Δ*nucS* derivative (blue), and the Δ*nucS* strain complemented with *nucS* from M. abscessus (Δ*nucS*/*nucS*_Mab_; orange). Error bars represent 95% confidence intervals, and asterisks indicate statistical significance (*P* < 10^−4^ in all cases, likelihood ratio test under Luria-Delbrück model, with Bonferroni correction).

M. abscessus infections are usually treated by complicated combinations of several antibiotics, due to the poor susceptibility of this pathogen to most antibiotics and the emergence of drug-resistant isolates (1, 19). Since combinations of macrolide-based (clarithromycin or azithromycin) and aminoglycoside (amikacin) antibiotics are very often used to treat M. abscessus (14, 16, 17), it was essential to analyze the acquisition of drug resistance through DNA mutations to both groups of antibiotics in a *nucS*-deficient M. abscessus strain (Fig. 3 and Table 1).

**TABLE 1:** Mutation rates of M. abscessus wild-type, Δ*nucS*, and complemented strain to clarithromycin, amikacin, ciprofloxacin, gentamicin, and apramycin resistance^*a*^

Antibiotic	Mutation rate [95% CI]		
	WT (ATCC 19977)	Δ*nucS*	Δ*nucS/nucS_Mab_*
Clarithromycin	1.50 × 10^−9^ [(0.80–2.41) × 10^−9^]	1.54 × 10^−8^ [(1.04–2.08) × 10^−8^]	1.57 × 10^−9^ [(0.91–2.39) × 10^−9^]
Amikacin	1.54 × 10^−8^ [(1.03–2.11) × 10^−8^]	2.62 × 10^−7^ [(2.02–3.21) × 10^−7^]	4.84 × 10^−8^ [(3.58–6.15) × 10^−8^]
Ciprofloxacin	≤2.00 × 10^−10^	4.46 × 10^−9^ [(3.21–5.85) × 10^−9^]	≤2.00 × 10^−10^
Gentamicin	9.25 × 10^−9^ [(6.44–12.3) × 10^−9^]	1.55 × 10^−7^ [(1.32–1.77) × 10^−7^]	5.06 × 10^−9^ [(3.39–6.91) × 10^−9^]
Apramycin	6.78 × 10^−9^ [(4.32–9.60) × 10^−9^]	1.51 × 10^−7^ [(1.28–1.74) × 10^−7^]	6.72 × 10^−9^ [(4.67–8.89) × 10^−9^]

a Rates of spontaneous mutations conferring antibiotic resistance of M. abscessus ATCC 19977 (wild-type, WT), Δ*nucS*, and Δ*nucS* complemented with the wild-type *nucS* gene from M. abscessus (Δ*nucS*/*nucS_Mab_*). Each value indicates the mutation rate to each antibiotic, with its 95% confidence interval in brackets.

First, MICs of the first-line antibiotics were evaluated in M. abscessus by using clarithromycin and amikacin as a representative macrolide and aminoglycoside, respectively. M. abscessus wild-type and Δ*nucS* strains showed a similar high susceptibility to both antibiotics tested, with MICs of 1 μg/mL (clarithromycin) and 4 μg/mL (amikacin) for both strains. These results indicated that *nucS* deletion did not increase the MICs to the antibiotics tested.

The rate at which resistance-conferring mutations were produced was measured in M. abscessus wild type and the *nucS*-null derivate by using clarithromycin (100 μg/mL) and amikacin (100 μg/mL). Both selected drug concentrations were above the susceptibility breakpoints proposed for testing rapidly growing mycobacteria (40). M. abscessus is considered drug resistant when the MICs of clarithromycin and amikacin are higher than 8 μg/mL or 64 μg/mL, respectively (40).

The acquisition rates of resistance to macrolides and aminoglycosides were evaluated by fluctuation tests (see Materials and Methods). The M. abscessus wild type had low rates of drug resistance acquisition that were higher with aminoglycosides (~10^−8^ to amikacin) than with macrolides (~10^−9^ to clarithromycin). The *nucS*-deficient strain exhibited a mutator phenotype with an increased rate of mutational resistance to both antibiotics, compared with the wild-type strain (Fig. 3 and Table 1), with a 10-fold increase to clarithromycin (1.54 × 10^−8^ for the Δ*nucS* strain versus 1.50 × 10^−9^ for the wild-type strain) and 17-fold to amikacin (2.62 × 10^−7^ for the Δ*nucS* strain versus 1.54 × 10^−8^ for the wild-type strain). Basal mutation rates were restored when the complemented strain was analyzed using both antibiotics. Therefore, inactivation of the noncanonical MMR led to increased emergence of drug resistance by mutation to the two front-line antibiotics used for treatment of M. abscessus infections.

### Analysis of drug resistance mutations in M. abscessus to first-line antibiotics.

To identify the type of point mutations that arose in M. abscessus wild-type and *nucS*-deficient strains, a set of independent drug-resistant colonies from both strains was analyzed by PCR amplification and sequencing of the target rRNA genes.

**FIG 4:**
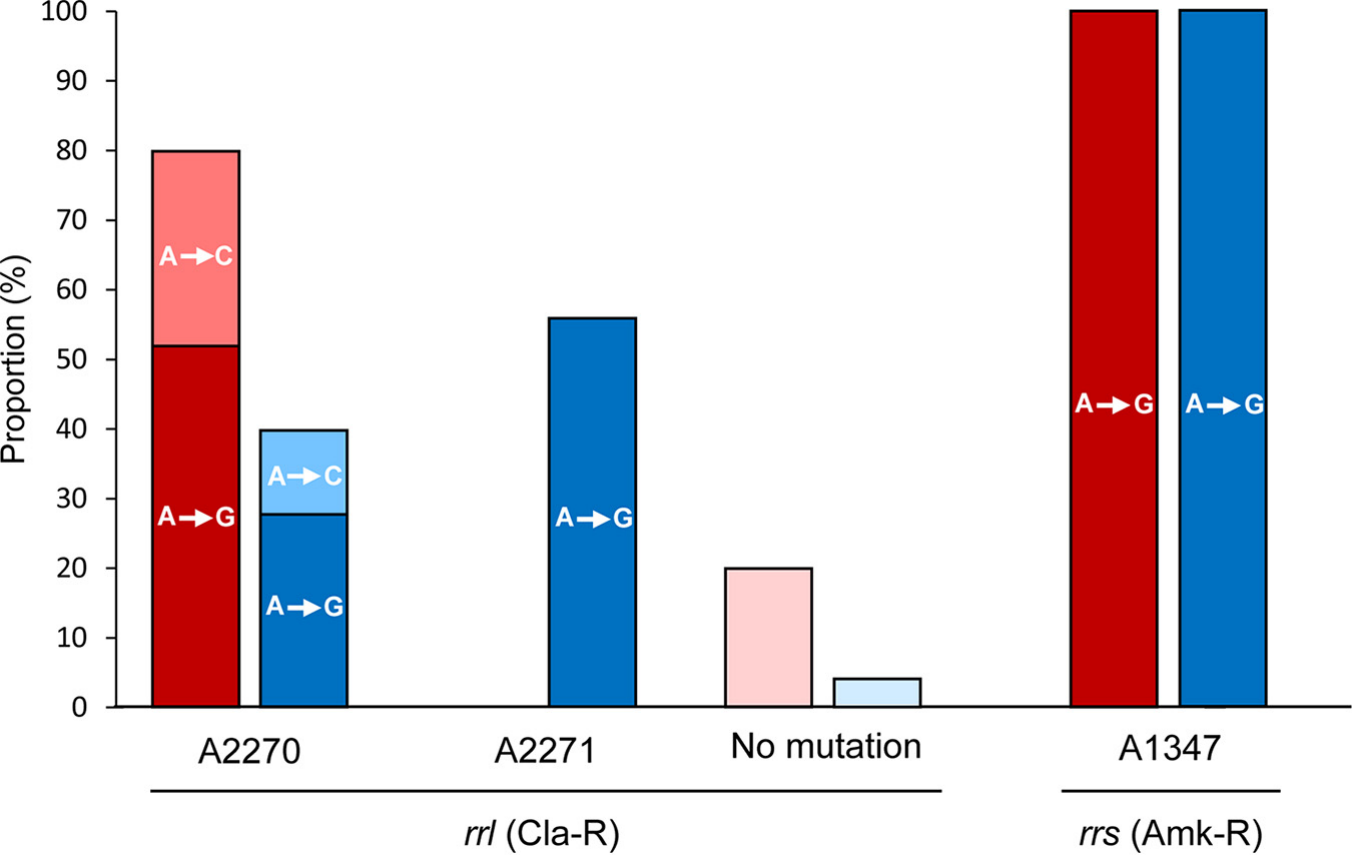
M. abscessus drug resistance mutations. Proportions of acquired mututations in the *rrl* gene conferring resistance to clarithromycin (Cla-R) (left) and in the *rrs* gene conferring resistance to amikacin (Amk-R) (right) of M. abscessus ATCC 19977 (wild-type [WT]; red) and Δ*nucS* (blue) are shown. Positions in each target gene are also indicated with the detected mutation in each bar. Color code: dark colors, transitions; light colors, transversions; very light colors, no mutation (unidentified mutation). A likelihood ratio test was used to compare the distribution of Cla-R mutations between the wild-type and Δ*nucS* strains (*P* < 10^−4^).

Acquired resistance to macrolides in M. abscessus clinical strains commonly emerges by 23S rRNA mutations (*rrl* gene) (20, 21). Analysis of the *rrl* gene in clarithromycin-resistant colonies (Cla-R) generated by the wild-type and Δ*nucS* strains revealed that 80% (20/25) of isolates derived from wild type and 96% (24/25) of Δ*nucS*-derived isolates contained *rrl* mutations (Fig. 4). In Cla-R isolates derived from the wild-type strain, the most commonly identified mutations were detected at position 2270 in the *rrl* gene (Escherichia coli *rrl* position 2058), with an A-to-G change at position 2270 (A2270G) (52% [13/25]) and a A2270C mutation (28% [7/25]). By contrast, the predominant mutation in Δ*nucS*-derived Cla-R isolates was at position 2271 in *rrl* (E. coli *rrl* 2059), with A2271G (56% [14/25]), while some mutations were also detected at position 2270 (28% [7/25] with A2270G and 12% [3/25] with A2270C) (Fig. 4). Differences in the distribution of Cla-R mutations (position and type of mutation) between the wild-type and the Δ*nucS* strain were significant (*P* < 10^−4^) according to a likelihood ratio test, indicating a shift in the mutational profile. A few Cla-R colonies showed no *rrl* mutations (20% [5/25] in wild-type colonies and 4% [1/25] in Δ*nucS* colonies) within the sequenced region, suggesting that other *rrl* mutations occurred outside the sequenced region (or within a different gene).

Constitutive macrolide resistance allowed us to determine the mutation spectra in both M. abscessus strains (Fig. 4). Once the total number of *rrl* mutations was analyzed, a higher proportion of transitions (A to G) were detected in Δ*nucS*-derived Cla-R isolates (87.5% [21/24]) than in the wild-type–derived ones (65% [13/20]) (*P* < 0.05), as expected in an MMR-deficient strain (31, 41). Transversions (A to C) tended to be slightly more common in Cla-R wild-type–derived colonies than in Δ*nucS*-derived Cla-R ones (35% [7/20] versus 12.5% [3/24]), but without statistical significance (*P* = 0.09).

Aminoglycoside resistance in M. abscessus clinical isolates is frequently associated with 16S rRNA mutations (*rrs* gene) (22). Characterization of the *rrs* gene in amikacin-resistant colonies (Amk-R) derived from the wild-type and Δ*nucS* strains indicated that all of them harbored an *rrs* mutation (100% [25/25] for both strains) (Fig. 4). The 16S rRNA gene (*rrs*) contained the same mutation at position 1374, *rrs* A1374G (E. coli 1408 *rrs* 1408) in all the wild-type– and Δ*nucS*-derived amikacin-resistant isolates (Fig. 4), a mutation previously identified as the main cause of amikacin resistance in M. abscessus (22).

The sequencing results supported that constitutive resistance to first-line antibiotics (macrolides and aminoglycosides) in M. abscessus is associated with genetic mutations at the target genes (rRNA genes) and may be driven by *nucS* deficiency.

### Extended range of drug resistance acquisition in M. abscessus by *nucS* inactivation.

**FIG 5:**
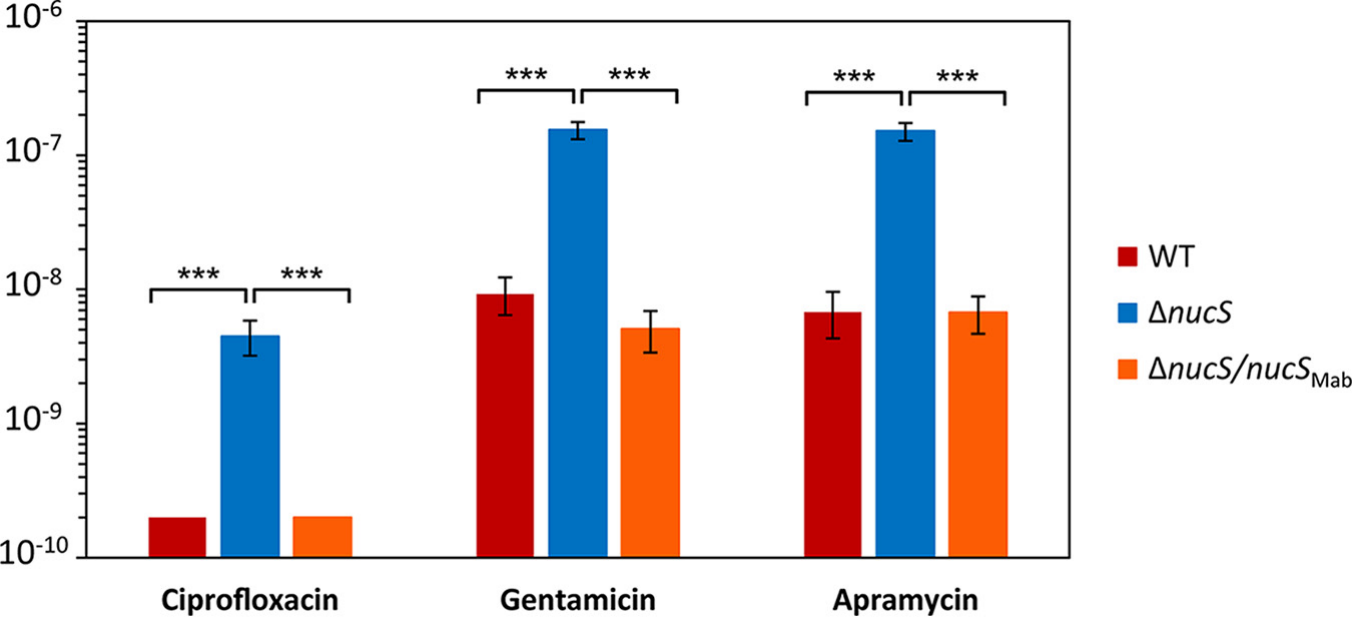
M. abscessus mutation rates to ciprofloxacin, gentamicin, and apramycin. Rates of spontaneous mutations conferring drug resistance of M. abscessus ATCC 19977 (wild type [WT]; red), its Δ*nucS* derivative (blue), and the Δ*nucS* strain complemented with *nucS* from M. abscessus (Δ*nucS*/*nucS*_Mab_; orange) are shown. Error bars represent 95% confidence intervals, and asterisks denote statistical significance (*P* < 10^−4^ in all cases, likelihood ratio test under Luria-Delbrück model, with Bonferroni correction).

To examine whether *nucS* deficiency led to a general mechanism of increased acquisition of drug resistance by mutation, three additional antibiotics were tested: two additional aminoglycosides, gentamicin (50 μg/mL) and apramycin (100 μg/mL), as well as the fluoroquinolone ciprofloxacin (50 μg/mL), a different class of antibiotic used in combination for alternative treatment of M. abscessus infections (Fig. 5 and Table 1).

In the aminoglycoside group, gentamicin is an active but less-used antibiotic against M. abscessus (25), while apramycin has been restricted to veterinary use (42), although it has been proposed as an alternative to amikacin due to its potent bactericidal activity (43). For both of the additional aminoglycosides tested, the rates of drug resistance acquisition were low and close to that detected for amikacin in the wild-type strain. Fluctuation test results confirmed that the Δ*nucS* strain displayed an enhanced emergence of drug resistance by mutation, with an ~16-fold increase observed with gentamicin (rate of 1.55 × 10^−7^ Δ*nucS* versus 9.25 × 10^−9^ wild type) and ~22-fold with apramycin (1.51 × 10^−7^ Δ*nucS* versus 6.78 × 10^−9^ wild type), within the same range previously detected with amikacin (Fig. 5 and Table 1). This event supported that the increase in mutation rate driven by noncanonical MMR inactivation affects the acquisition of resistance to widely used agents for clinical treatments (amikacin) as well as antibiotics with less (gentamicin) or no (apramycin) clinical utility for the treatment of M. abscessus infections in humans.

In the case of fluoroquinolones, several compounds (ciprofloxacin, moxifloxacin, and levofloxacin) have shown good activity against M. abscessus, and they are often included in multiple-drug therapy to improve efficacy (18, 44). Fluoroquinolones are also alternative agents for the treatment of M. abscessus infections when drug resistance to the front-line antibiotics arises. When the fluoroquinolone ciprofloxacin was tested in M. abscessus, the wild-type strain did not develop detectable resistance to ciprofloxacin (≤2.00 × 10^−10^). By contrast, a significant rate of spontaneously emerging ciprofloxacin resistance (4.46 × 10^−9^) was observed with the Δ*nucS* strain (Fig. 5 and Table 1). This result suggests that inactivation of noncanonical MMR could be a risk for the emergence of drug-resistant isolates that would otherwise remain unnoticeable.

Therefore, according to the fluctuation test results with all antibiotics tested, we can conclude with confidence that the inactivation of *nucS* generated a hypermutator phenotype in M. abscessus. In summary, we propose a key role of the NucS-dependent noncanonical MMR in suppression of mutations that are able to confer resistance to multiple groups of antibiotics, including some of the few that are available for treatment of M. abscessus infections.

## DISCUSSION

M. abscessus is a major clinical concern due to its ability to produce severe chronic diseases, and it is the pathogen with the worst impact on lung function in patients with CF (45). M. abscessus treatments are complex, with limited success, and require a combination of multiple drugs based on macrolides (clarithromycin or azithromycin) and aminoglycosides (amikacin), plus alternative second-line antibiotics, including fluoroquinolones (14).

Acquisition of genomic mutations plays a key role in how this pathogen infects and adapts to a susceptible host. Evolutionary changes that promote established infections in the CF lung involve within-host mutations in a small set of genes leading to increased virulence and drug resistance (46). Mutations have resulted in the generation of a wide genetic diversity, with different subclones within the same patient possibly showing different antibiotic susceptibility profiles (46, 47), a common trait developed by other chronic lung pathogens in patients with CF (48–51).

The MMR pathway plays a major role in preventing mutations that could lead to antibiotic resistance in pathogenic bacteria and to the evolution of the infection (28, 52). While it has been previously established that a NucS-dependent noncanonical MMR mechanism ensures genome stability and prevents mutations (30, 31, 34, 35), our work here highlights for the first time its impact on the rise of acquired drug resistance in a bacterial pathogen, M. abscessus. A plethora of drug resistance mechanisms can be displayed by this pathogen, including mutations that modify the specific drug target driving constitutive resistance (1, 19). The emergence of further drug resistance is a challenge for treatment success against M. abscessus infections.

NucS activity in genome maintenance is essential for the efficacy of frontline antibiotics (macrolides, aminoglycosides, and fluoroquinolones) against this pathogen. When mutations were not prevented due to *nucS* inactivation, the rate of acquisition of drug resistance was significantly increased, and this could facilitate the emergence and spread of M. abscessus drug-resistant strains. The mutator phenotype exhibited by inactivation of M. abscessus *nucS* generated resistant mutants with rates ranging from a 10-fold increase with macrolides (clarithromycin) to a 16- to 22-fold increase with aminoglycosides (amikacin, gentamicin, and apramycin). Furthermore, resistance to fluoroquinolones (ciprofloxacin) developed at low but detectable levels in the *nucS*-null strain but not in the wild-type strain. Differences in estimated mutation rates between M. abscessus wild-type and *nucS-*deficient strains were remarkable but lower than those reported in other nonpathogenic actinobacteria, including M. smegmatis (30) and Corynebacterium glutamicum (34). However, it is important to consider that M. abscessus also has inducible mechanisms for development of resistance to macrolides and aminoglycosides that could overlap and partially mask the acquisition of drug resistance mutations (23, 25).

Macrolide and aminoglycoside resistance can be achieved though rRNA gene mutations (23S rRNA and 16S rRNA, respectively) (20–22). Sequence analysis of antibiotic-resistant isolates (clarithromycin- and amikacin-resistant derivates) revealed the mutational profile in M. abscessus wild-type and Δ*nucS* strains. Constitutive macrolide resistance in wild-type–derived M. abscessus isolates was conferred by *rrl* mutations that emerged with A2270G/C changes, the most common mutations associated with macrolide resistance in M. abscessus clinical isolates (4). Interestingly, a different mutation, *rrl* A2271G, was the most prominent variant conferring clarithromycin resistance in Δ*nucS*-derived isolates. A transition-biased mutational spectrum in M. abscessus Δ*nucS*, as seen in MMR-deficient strains (41), was observed in macrolide-resistant *rrl* variants derived from M. abscessus Δ*nucS*. For aminoglycosides (amikacin), very few *rrs* positions are modified by mutation to generate constitutive resistance (4, 22), with *rrs* A1374G reported as the only change associated with amikacin resistance in M. abscessus clinical strains (4). Indeed, all amikacin-resistant isolates generated from both M. abscessus wild-type and Δ*nucS* strains contained the same A1374G mutation. Ciprofloxacin-resistant isolates were not sequenced, as *gyrA* and/or *gyrB* mutations appear to be a controversial target for acquisition of fluoroquinolone resistance in M. abscessus (53, 54).

The majority of M. abscessus infections are generated by successful dominant clones that have expanded globally. These are associated with chronic infections, high levels of drug resistance, and poor clinical outcomes (3, 55). These dominant circulating clones frequently develop constitutive resistance to two key NTM antibiotics, aminoglycosides (amikacin) and macrolides, through point mutations (16S and 23S rRNA) (3). Because extended antibiotic pressure acts as the main selective force in the spread of resistant strains (56), mutational resistance could be driven and/or accelerated by DNA repair deficiencies, as illustrated in the M. abscessus *nucS*-null strain.

It has been shown that hypermutator strains can evolve and succeed during chronic infection under long-term antibiotic pressure (27, 48, 49). Genetically diverse M. abscessus populations generated by mutations can adapt quickly during chronic lung infections (47). Considering also its impact on the acquisition of drug resistance, it may be interesting to determine whether some M. abscessus clinical isolates carry DNA repair deficiencies (such as *nucS* polymorphisms), as seen in other chronic lung pathogens (27, 48–51). M. abscessus clinical strains with hypermutator phenotypes were recently isolated in patients with CF, since DNA repair deficiencies could accelerate within-host evolution and genetic diversification of this pathogen (46). Interestingly, a bioinformatics analysis of a data set of genomic assemblies allowed us to determine that some M. abscessus clinical strains are polymorphic, carrying the same substitution (NucS T74I) (Fig. 1).

NucS analysis could be a valuable predictive marker to evaluate the potential success of drug treatments against M. abscessus clinical isolates, in combination with established antibiotic resistance markers evaluated by genotyping (40, 57–59). It may also be interesting to explore its potential role in bacterial virulence, because M. abscessus requires genetic mutations for the transition from smooth to hypervirulent rough colonies (60), as these variants dominate during severe and persistent forms of M. abscessus chronic infection (1).

## MATERIALS AND METHODS

### Bacterial strains and growth conditions.

M. abscessus ATCC 19977 strain (wild type) (Mycobacterium abscessus subsp. *abscessus*) and its derivatives were grown in LB broth, Middlebrook 7H9 broth, or Middlebrook 7H10 agar plates (Difco), with 0.5% glycerol, 0.5% Tween 80, and 10% oleic acid-albumin-dextrose-catalase and supplemented with antibiotics when required (50 μg/mL kanamycin or 50 μg/mL zeocin). M. abscessus liquid cultures were grown in flasks with shaking (250 rpm) at 37°C. For solid cultures, all plates were incubated for 5 to 7 days at 37°C to obtain M. abscessus colonies. All cloning was performed in Escherichia coli DH5α grown in LB supplemented with antibiotics when appropriate (50 μg/mL kanamycin or 100 μg/mL zeocin).

### Cloning.

PCRs were performed using Q5 high-fidelity DNA polymerase (NEB) and M. abscessus genomic DNA as a template. For *nucS* deletion, a 5′ *nucS* DNA fragment was amplified by PCR using PciI5F (5′-CTGACATGTTCTCGTTCGCGCAGGCCCTCCACCA-3′) and NotI5R (5′-ATTGCGGCCGCGCACTGAGCAACGACGAGACGCAC-3′) oligonucleotides, while a 3′ *nucS* DNA fragment was generated by PCR with PacI3F (5′-GGATTAATTAATCGGACGAGTTCCGATTGTTCTGA-3′) and SpeI3R (5′-GAGACTAGTCCCCATGATCACGTAATCCACTGCG-3′) oligonucleotides. For *nucS* complementation, full-length *nucS* was produced by PCR using EcoF (5′-CGAGAATTCGCGAATCGACTCGTTTGCCAT-3′) and HindIIIR (5′-CGAAAGCTTTCAGAACAATCGGAACTCGTC-3′) primers. PCRs, DNA digestions with restriction enzymes (NEB), and DNA ligations with T4 DNA ligase (NEB) were performed following the manufacturer’s instructions.

### Generation of a Δ*nucS* knockout mutant in M. abscessus.

The M. abscessus Δ*nucS* (MAB_1460, *nucS_Mab_*) deletion mutant was generated by recombineering (38). Two DNA fragments (5′ *nucS* and 3′ *nucS*) that flanked the target gene were PCR amplified (see above). Then, they were cloned into the pSGV53 vector (39), upstream and downstream of a zeocin resistance gene, to construct a plasmid that contained the *nucS* deletion (pSGV-Δ*nucS zeoR*). The full cassette harboring the *nucS* gene deletion was amplified by PCR using PciI5F and SpeI3R primers and transformed into M. abscessus ATCC 19977 containing pJV53 plasmid (38). Insertion of the deletion cassette by a double-crossover event through recombination was selected using 50 μg/mL zeocin to generate the Δ*nucS* strain.

### Construction of a complemented strain in M. abscessus.

The M. abscessus Δ*nucS* deletion mutant was complemented using a full-length *nucS_Mab_* gene cloned into pMV361 integrative vector (61) and introduced into the deletion mutant. For complementation, *nucS*, including its upstream region with its own native promoter, was PCR amplified, and the resulting PCR product was digested and cloned into a pMV361 backbone, to generate pMV361-*nucS_Mab_*. The complementation plasmid was electroporated into the M. abscessus Δ*nucS* deletion mutant and inserted by site-specific recombination at the *att* site in M. abscessus to generate the complemented strain.

### PCR confirmation of constructed strains.

To check and verify the constructed strains, PCRs were performed to identify each specific M. abscessus strain. First, extracted genomic DNA samples were tested for *nucS* amplification (positive in the wild-type and complemented strains, negative in the Δ*nucS* deletion mutant) with *nucS*F (5′-ACCGTTAATTATGTTGGCCGGCTG-3′) and *nucS*R (5′-CGAGTCCATTCCCCGCATAGCGTC-3′) primers. After that, samples were tested for PCR amplification of the zeocin resistance gene (positive in the Δ*nucS* deletion mutant and complemented strains, negative in the wild-type strain) with *zeo*F (5′-TACGACAAGGTGAGGAACTAAACC-3′) and *zeo*R (5′-CCCCAATTAATTTCAGTCCTGCTC-3′) primers. The specific PCR amplification pattern of each M. abscessus strain is shown in Fig. 2B.

### Bacterial growth and viability.

Bacterial growth was evaluated by growth curves of M. abscessus wild-type (ATCC 19977) and Δ*nucS* strains. Cultures (in quadruplicate) were inoculated with an initial optical density at 600 nm (OD_600_) of 0.05 in Middlebrook 7H9 broth and incubated 72 h at 37°C with shaking. Bacterial growth was evaluated by measuring the OD_600_ for each culture every 4 h. Cell viability was analyzed by plating the cultures in Middlebrook 7H10 agar and counting the number of bacterial colonies (CFU) per milliliter after 7 days at 37°C.

### Measurement of mutation rates.

Mutation rates to each antibiotic were determined by fluctuation tests and validated by at least three different experiments each, as previously described (30). Briefly, a starting culture of each strain was diluted 1:1,000 to generate 8 to 10 independent liquid cultures in Middlebrook 7H9 broth and incubated 3 to 5 days with shaking (250 rpm) at 37°C. All the cultures were treated with glass beads and shaked vigorously for 1 min to break the clumps before using them for the evaluation of the mutation rates in each strain. All the grown cultures (10^9^ to 10^10^ cells/mL) and serial dilutions were plated on Middlebrook 7H10 agar plates without antibiotic (for viable cells) and with antibiotic (for drug-resistant cells) and incubated for 7 to 10 days at 37°C. After this incubation, CFU were counted to obtain the total number of viable cells and mutant cells in the cultures.

The expected number of mutations per culture and 95% confidence intervals were evaluated using the maximum likelihood estimator, as previously described (30, 62). In brief, to calculate the expected number of mutations that occur in each culture (*m*) and its 95% confidence intervals, the functions “newton.LD.plating” and “confint.LD.plating,” which account for plating efficiency, were used. Mutation rates were calculated by dividing *m* by the final number of cells, which was taken as an estimator of the total number of generations. Finally, statistical comparisons were performed by the likelihood ratio test method using the LRT.LD.plating function, which accounts for plating efficiency and differences in the final numbers of cells. When performing multiple comparisons, Bonferroni correction was applied to *P* values. All functions used were implemented in the package rSalvador for R (http://eeeeeric.com/rSalvador/) (62).

### MIC determinations.

To evaluate MICs, M. abscessus strains were grown in 7H9 broth until they reached stationary phase (48 h at 37°C). The cultures were diluted (1:1,000) and were inoculated in 96-well plates containing serial 2-fold dilutions of antibiotics in 7H9 broth (0.1-mL volume) and incubated 48 h at 37°C. The MICs were evaluated and confirmed by the addition of resazurin (30 μL from a 200-μg/mL stock) to the plates.

### Sequencing of drug-resistant isolates.

rRNA genes (23S *rrl* and 16S *rrs*) were PCR amplified from independent clarithromycin- and amikacin-resistant isolates (25 isolates per strain) and sequenced to identify the type(s) of resistance mutations. For 23S *rrl*, it was PCR amplified using a pair or primers (23SrRNAF, 5′-TGCTGGAAGGTTAAGAGGACC-3′; 23SrRNAR, 5′-CCTTGTGCACTTGCACTCAAC-3′). 16S *rrs* was PCR amplified using two specific primers (16SrRNAF, 5′-TATGTTGCCAGCGGGTAATGC-3′; 16SrRNAR, 5′-AGGTGATCCAGCCGCACCTTC-3′). PCR products were sequenced and compared to rRNA gene reference sequences to identify the type of point mutation in each drug-resistant isolate.
