# Supplementary material for: Noncanonical Mismatch Repair Protein NucS Modulates the Emergence of Antibiotic Resistance in Mycobacterium abscessus
Source: Microbiol Spectr. 2022 Oct 11;10(6):e02228-22. doi: 10.1128/spectrum.02228-22 (PMC9769700; doi:10.1128/spectrum.02228-22)
Supplement: Supplemental file 1 — Supplemental material. Download spectrum.02228-22-s0001.pdf, PDF file, 0.1 MB [file spectrum.02228-22-s0001.pdf]

Supplementary Figure 1.

A)

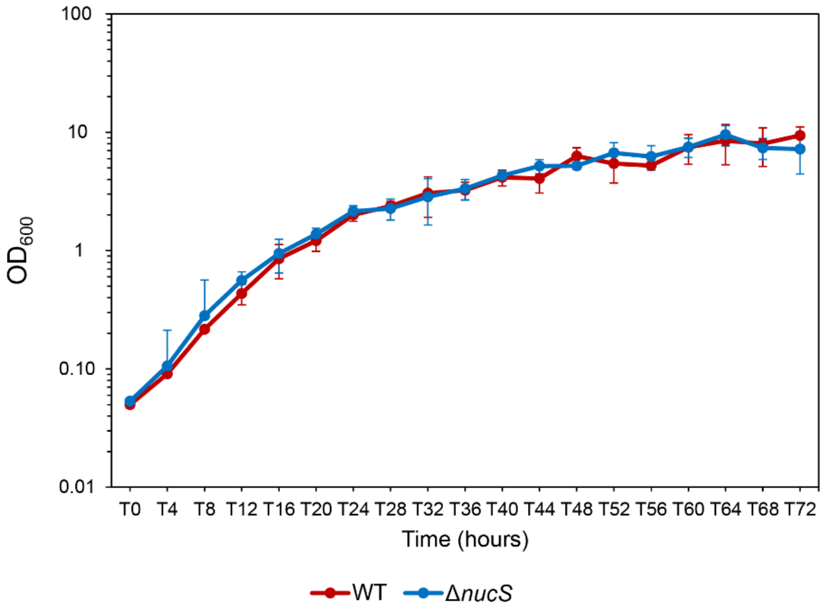

B)

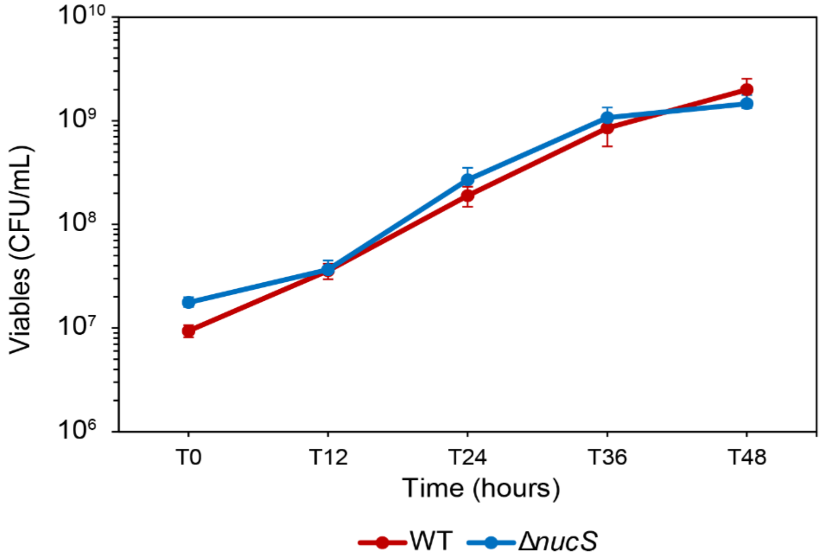

Supplementary Figure 1. Bacterial growth and viability. A) Growth curve depicting OD_{600} of the *M. abscessus* wild-type and Δ*nucS* cultures in Middlebrook 7H9 broth. B) Viability assay with the number of viable cells per mL (CFU/mL) of the *M. abscessus* wild-type and Δ*nucS* cultures plated in Middlebrook 7H10 agar.
